# Supplementary material for: High-speed mapping of surface charge dynamics using sparse scanning Kelvin probe force microscopy
Source: Nat Commun. 2023 Nov 8;14:7196. doi: 10.1038/s41467-023-42583-x (PMC10632481; doi:10.1038/s41467-023-42583-x)
Supplement: Supplementary file 1 — Supplementary Information [file 41467_2023_42583_MOESM1_ESM.pdf]

Supplementary Information of: High-speed mapping of surface charge dynamics via sparse scanning Kelvin probe force microscopy

Marti Checa[1*], Addis S. Fuhr[1], Changhyo Sun[2], Rama Vasudevan[1], Maxim Ziatdinov[1,3], Ilia Ivanov[1], Seok Joon Yun[1], Kai Xiao[1], Alp Sehirlioglu[4], Yunseok Kim[2], Pankaj Sharma[5,6], Kyle P. Kelley[1], Neus Domingo[1], Stephen Jesse[1], Liam Collins[1*]

[1]Center for Nanophase Materials Sciences, Oak Ridge National Laboratory, Oak Ridge, Tennessee 37831, United States.

[2]School of Advanced Materials Science and Engineering, Sungkyunkwan University, Suwon 16419, Republic of Korea.

[3]Computational Sciences and Engineering Division, Oak Ridge National Laboratory, Oak Ridge, TN 37923, USA.

[4]Department of Materials Science and Engineering, Case Western Reserve University, Cleveland, Ohio 44106, United States.

[5]College of Science and Engineering, Flinders University, Bedford Park, SA 5042, Australia.

[6]ARC Centre of Excellence in Future Low-Energy Electronics Technologies (FLEET), UNSW Sydney, NSW 2052, Australia.

Supplementary note 1. Comparison of AM-KPFM with H-KPFM

Amplitude modulated KPFM (AM-KPFM) in lift (2-pass) mode is the most common implementation of KPFM, mainly due to its easy implementation. However, as it is based in force detection instead of force gradient detection, its CPD accuracy suffers from non-local stray capacitance between cone, cantilever, and sample, resulting in a reduction of spatial resolution and quantitativeness. To solve that issue, Sugawara et al.[1] developed heterodyne KPFM (H-KPFM), which boosts short range force sensitivity reducing CPD crosstalk.

There are a few different implementations of force-gradient sensitive KPFM (see details of each implementation in [2]), but 2 of them are the most commonly used:

i) FM Sideband KPFM, where the frequency of electrical excitation is lower than the first eigenmode of the cantilever ($\omega_E < \omega_0$), while the detection is performed using the sidebands at $\omega_m \pm \omega_E$ with the mechanical oscillation frequency ω_m at the first eigenmode. In this implementation, ω_m should be sufficiently high to decouple the detection sidebands from the mechanical carrier, which turns into a decrease of the SNR as detection is performed further away from the resonance. Therefore, this mode

typically uses higher AC voltages and low detection bandwidths, limiting the speed of the measurement.

ii) FM Heterodyne KPFM, where the electrical excitation is performed at $\omega_{E}= \omega_{1}- \omega_{0}$, which shifts the sideband frequency to the second eigenmode of the cantilever ω_{1}. The main advantage of it being the use of resonance amplification, boosting SNR, enabling faster imaging speeds. This is the implementation we have used in this work, and it is also the implementation of the benchmark high-speed KPFM by Garret et al[3,4].

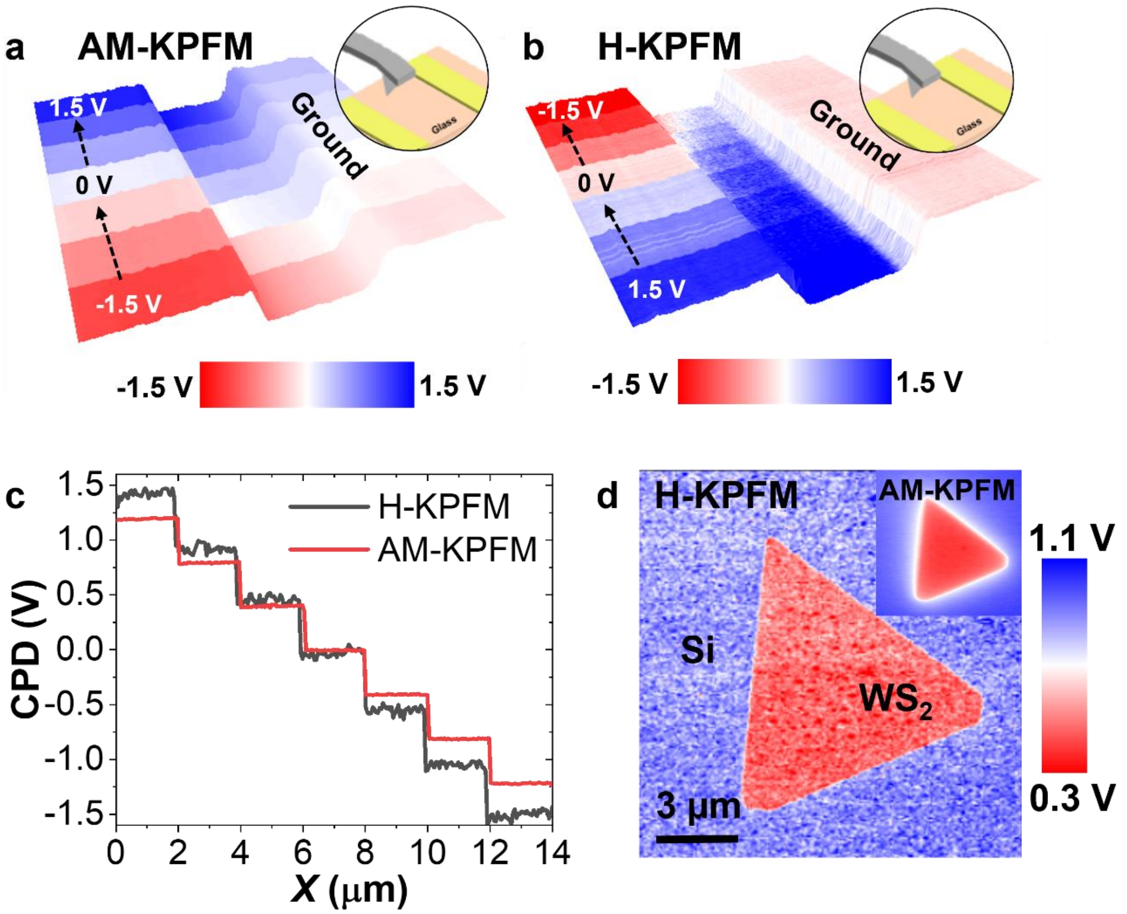

Supplementary Figure 1: a) AM-KPFM and b) H-KPFM of the interdigitated electrodes, applying an external voltage of ±1.5 V. c) Profiles of the AM-KPFM and H-KPFM images. d) H-KPFM of a WS_2 flake grown on a Si substrate. Inset is the AM-KPFM of the same region.

In Supplementary Figure 1 we show the implementation of AM-KPFM and H-KPFM onto the same sample (interdigitated electrodes deposited onto glass). CPD maps are acquired during DC bias application between the planar electrodes. Images (1a and 1b) show the superior locality of H-KPFM and profiles in 1c show how AM-KPFM fails to recover the ±1.5 V externally applied

due to the previously mentioned artifacts. Finally, in Figure 1d both techniques are also applied to image 2D WS_2 flakes deposited onto a silicon substrate (same sample as Figure 1 in the main manuscript), where we can see the increased spatial resolution of H-KPFM through the sharper edges of the flake as compared to the AM-KPFM image (1d Inset).

Supplementary note 2. Detailed schematics of the experimental setup

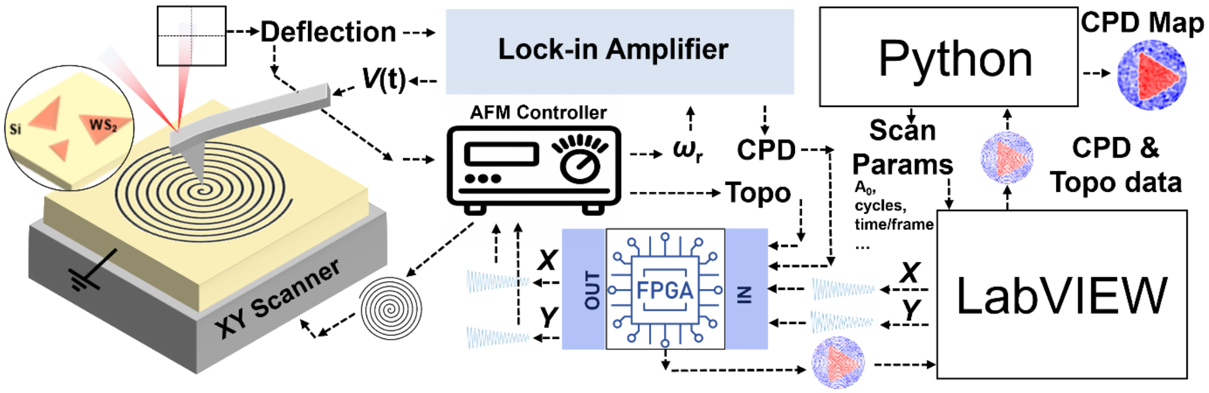

Supplementary Figure 2: Detailed schematic of the experimental setup for SS-KPFM: A python script sets up the scan parameters and sends it to LabVIEW, which generate the X and Y piezo volage signals and inputs it to the FPGA, which sends it to the XY scanner through the AFM controller. H-KPFM is running in parallel using an external Lock-in amplifier while the spiral scan path is being performed. CPD and topography are read by the FPGA and sent to LabVIEW. Finally, Python performs the GP processing on sparse scan data to obtain the final CPD Map.

Supplementary note 3. Lissajous curve scan example

For this work, we have focused on spiral trajectories, but one of the advantages of such implementation using FPGA driven control of the XY AFM piezo controllers is that it allows for tunability of scan path to any desired waveform. To show such capabilities, we have captured 2 additional images over the same region, in the WS_2 sample from Figure 1, one with spiral scan trajectory and the other one following a Lissajous curve (Supplementary Figure 3).

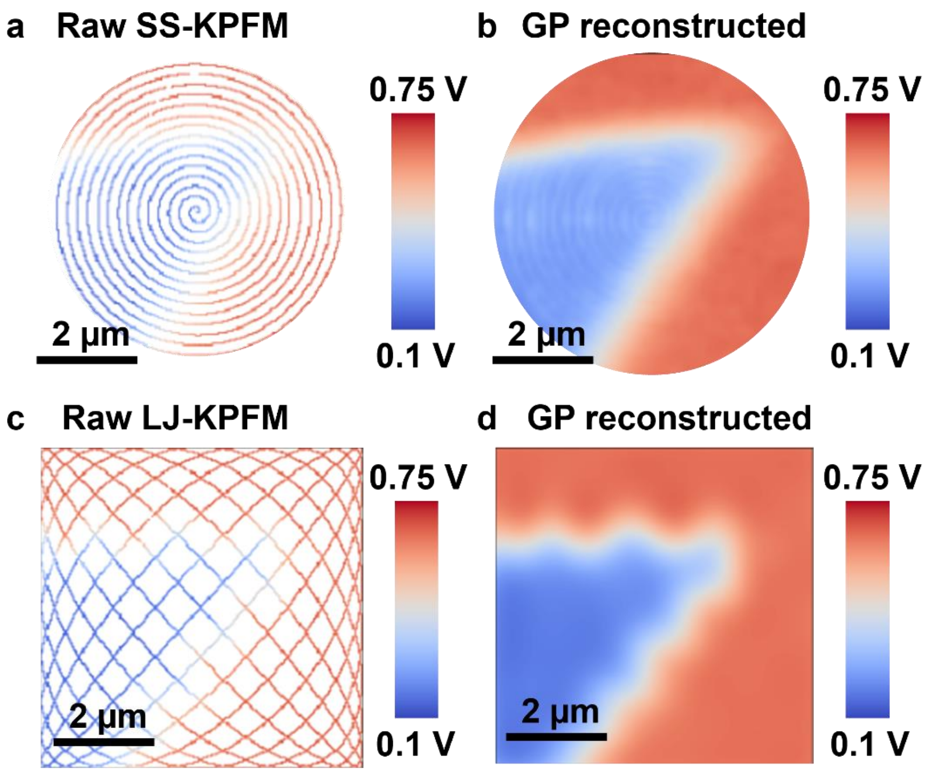

Supplementary Figure 3: a) Raw data of SS-KPFM image. b) Corresponding GP reconstruction. c) Raw data acquired using a Lissajous curve scan path in the same region as a. d) Corresponding GP reconstruction.

Overall, different sample geometries or structures, would result into different optimal tip trajectories for an optimized fast information capture. For instance, samples containing most relevant information close to corners of the scanned area, would be more benefitted by the Lissajous trajectories, whereas samples where information is confined in the center would benefit from spiral scans. Additionally, similarly to what happens with different sparsities, the hyperparameters of the inpainting algorithms (GP in this case), might have to be tunned again independently for each different scan path followed during the Raw data acquisition.

Supplementary note 4. SNR analysis

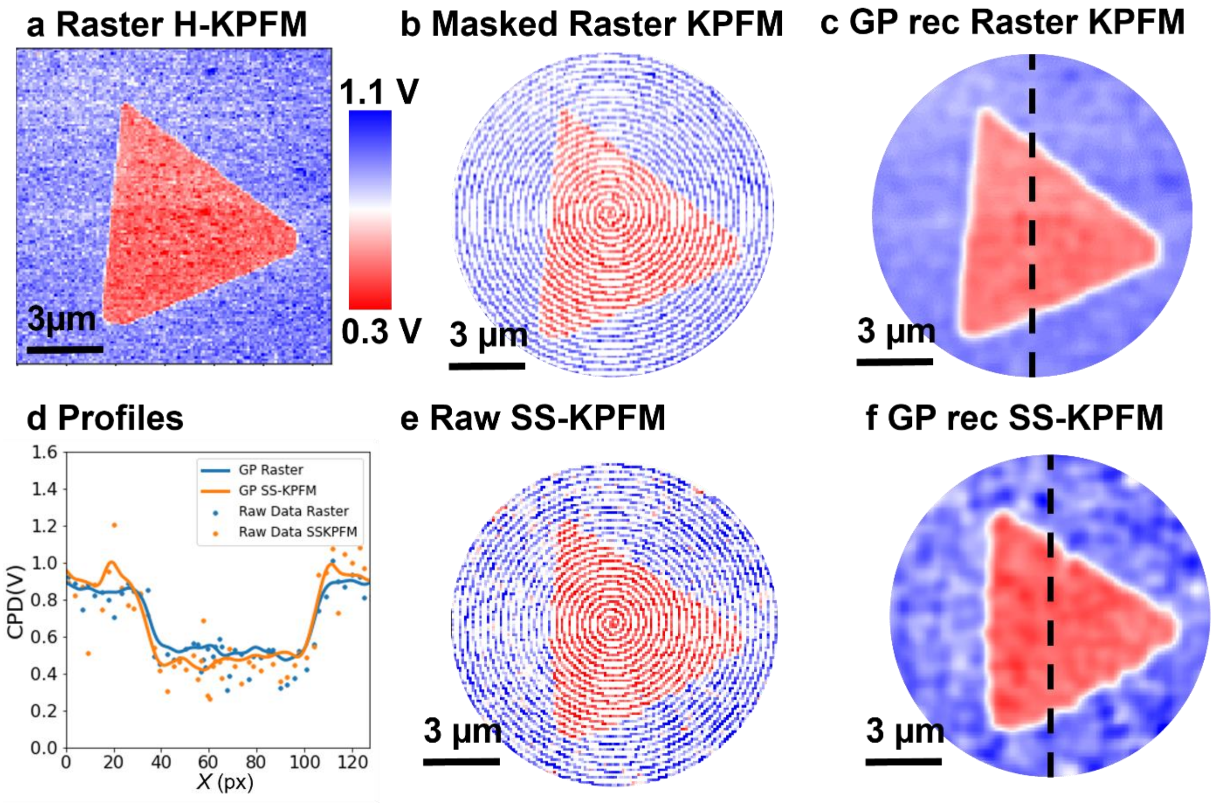

Supplementary Figure 4: a) Raw data of raster scan H-KPFM image. b) Masked raster scan H-KPFM image. c) GP reconstructed (rec) image in b. e) Raw data of SS-KPFM. F) GP reconstructed image in e. d) Profiles along the black dashed line.

Supplementary note 5. Correlation Length parameter for GP inpainting

In Supplementary Figure 5 we can see the effect of the correlation length for the GP algorithm. There is a compromise between spatial resolution and signal to noise ratio. Images inpainted using longer correlation length seem to have less spatial resolution and less noise appearing as more “blurred” but “smooth”, whereas images using shorter correlation length seem to have more spatial resolution, but also more noise.

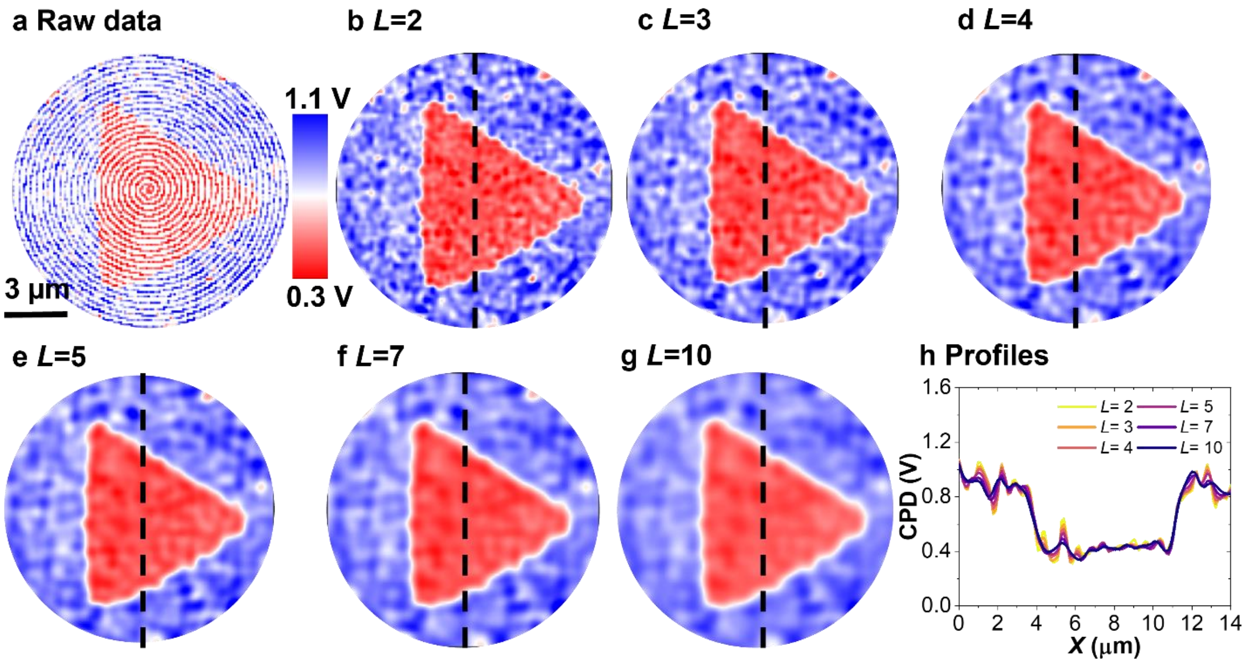

Supplementary Figure 5: a) Raw data for the 24 cycles spiral on the WS_2 triangular flake (same as in Figure 2 of the main manuscript). b) GP inpainting with L=2. c) GP inpainting with L=3. d) GP inpainting with L=4. e) GP inpainting with L=5. f) GP inpainting with L=7. g) GP inpainting with L=10. h) Profiles. All images have the same size (14µm radius) and colorbar.

Supplementary note 6. Topography of Figure 2

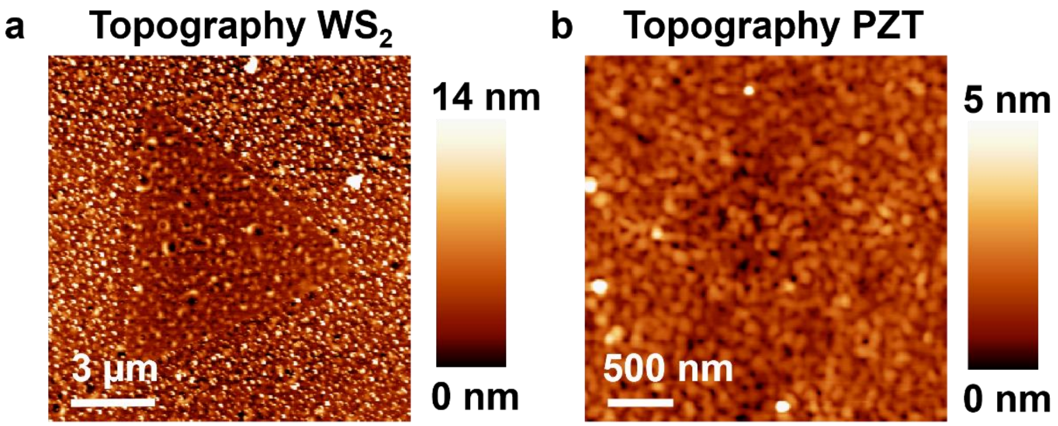

Supplementary Figure 6: a) Topography of WS_2 flake of Figure 2 of the main manuscript. b) Topography of PZT of Figure 2 of the main manuscript.

Supplementary note 7. Bulk impedance measurements

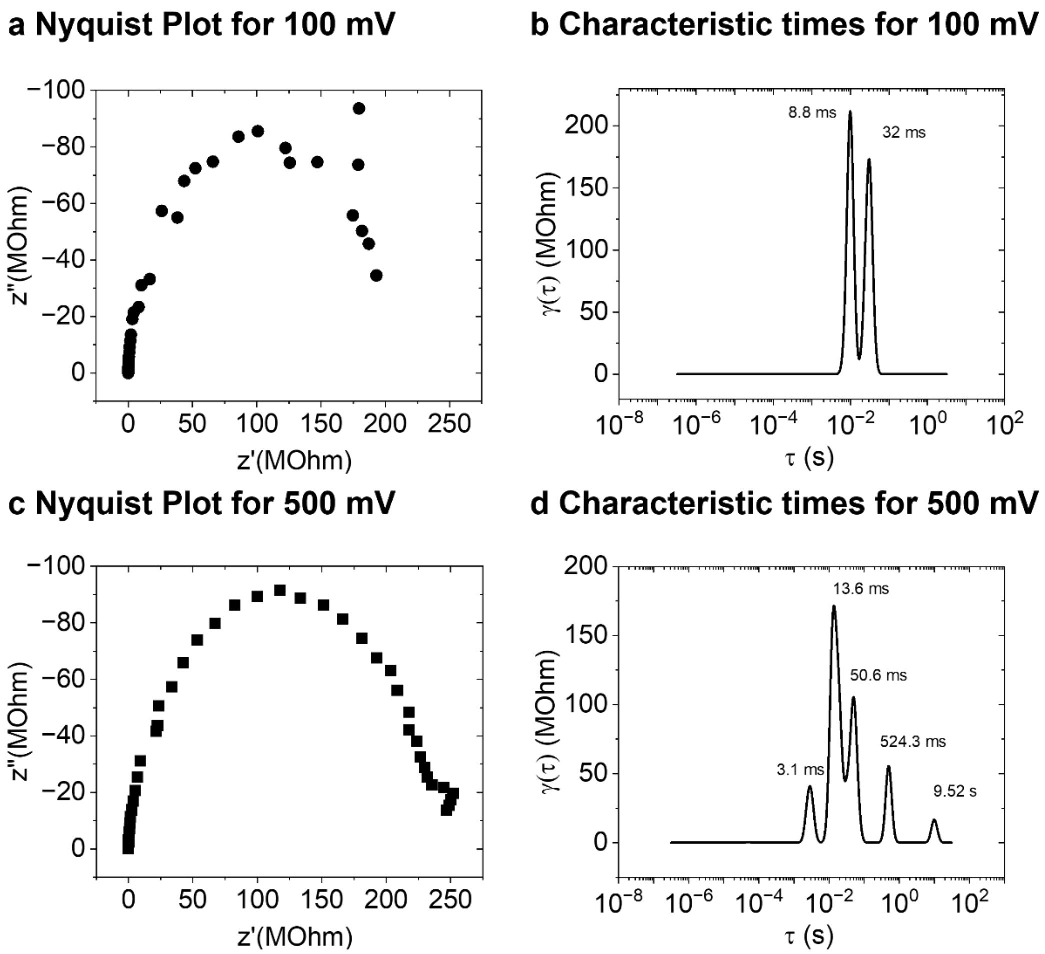

Supplementary Figure 7: a) Nyquist plot of the LAO/STO planar device for a 100 mV AC bias. b) Characteristic times of the device derived from a. c) Nyquist plot of the LAO/STO planar device for a 500 mV AC bias. d) Characteristic times of the device derived from c.

Supplementary note 8. CPD evolution with lateral DC bias

CPD signal shows that the long characteristic time (10 s range) is not activated for biases below 500 mV, where there is only a fast response (on the ms range). Which points towards the slower response being related to the activation of a faradaic process.

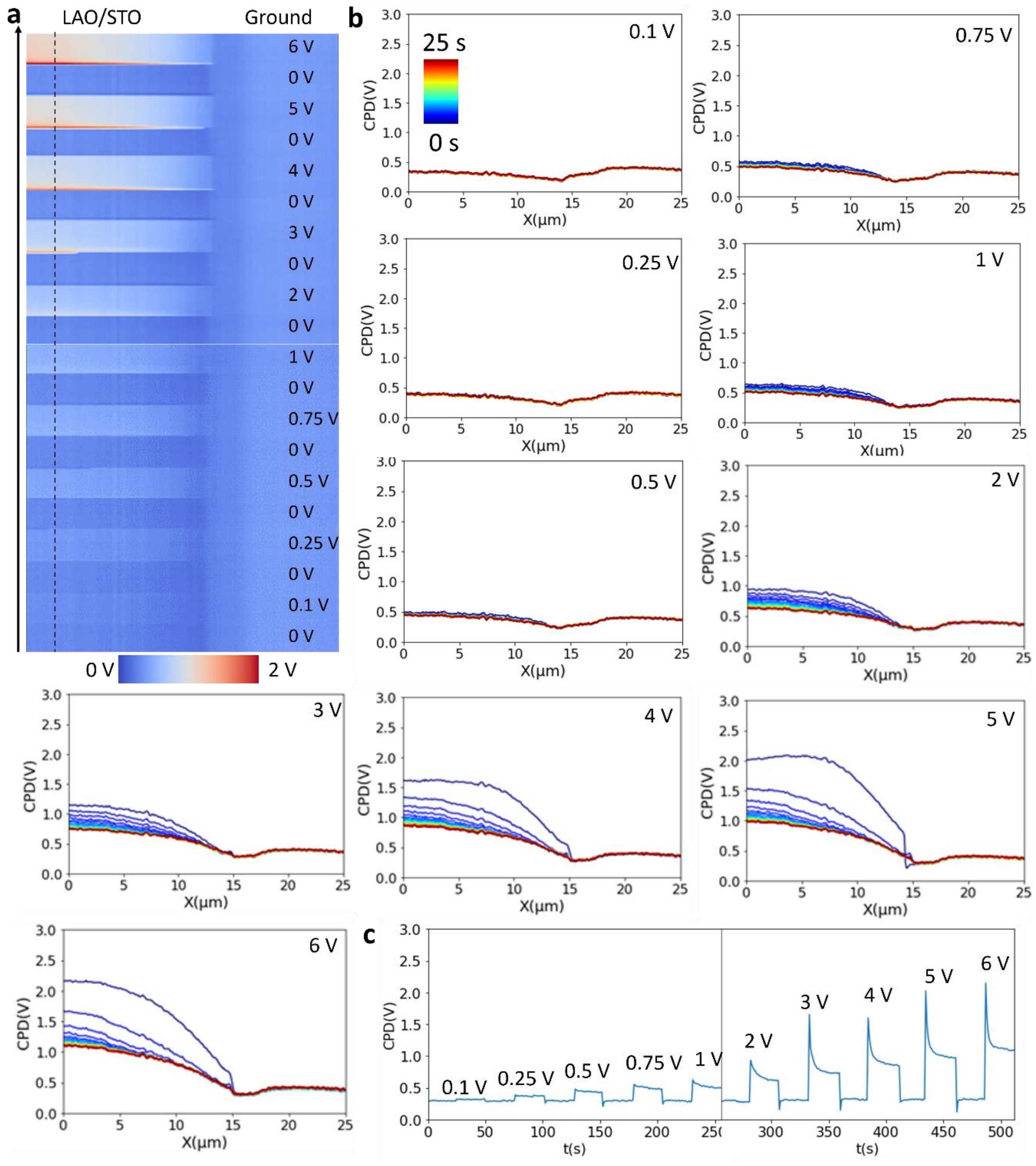

Supplementary Figure 8: a) H-KPFM CPD maps over the grounded electrode as a function of lateral DC bias from 0 V to +6 V. b) CPD line profiles over each different DC bias step. c) CPD temporal profile along a black dashed line in a.

Supplementary note 9. Bulk impedance measurements as a function of humidity

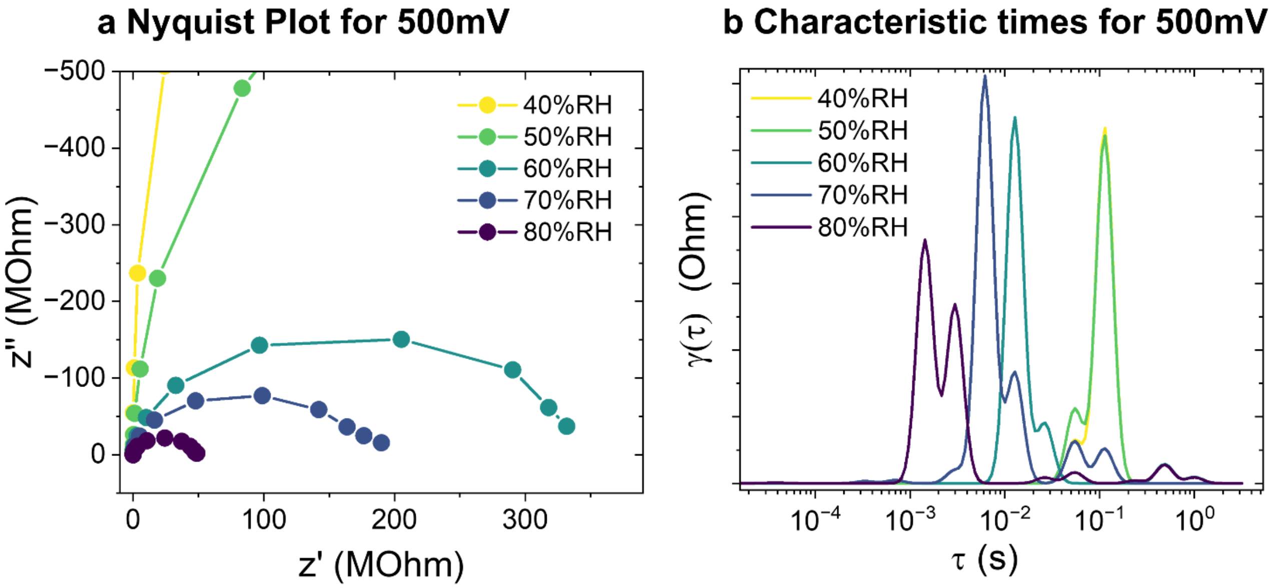

Supplementary Figure 9: a) Nyquist plot of the LAO/STO planar device for different RH. b) Characteristic times of the device derived from a.

Supplementary note 10. Charge injection/diffusion for different bias pulses

We observe a clear asymmetry in the CPD dynamics for both positive biases (Supplementary Figure 10e, 10f and 10g) and for negative ones (Supplementary Figure 10h, 10i and 10j). See also animations with all the CPD frames in Animation 3-8. Furthermore, the induced CPD change is positive for positive applied voltages and negative for negative ones. This means that the sign of the applied bias governs the charge injection mechanism[5], with oxygen ions (O^{-2}) being driven into the material for negative bias, reducing local conductivity, and oxygen vacancies being pulled out for positive bias, increasing local conductivity.

We also observed that the extent of the region impacted by charge injection depends on the magnitude of the DC pulses and is correlated with the surface topography. Higher biases lead to more charge injection/redistribution, resulting in larger CPD variations. However, an intriguing question arises regarding the relationship between charge injection/diffusion and the grains (or grain boundaries) present on the polycrystalline surface. By simultaneously acquiring topography and CPD maps, we can investigate the spatial correlation between the two parameters, as illustrated by the dashed lines (that highlight the grain boundaries) in Supplementary Figure 10. This information can provide valuable insights into the behavior of charges on the surface and their interaction with the underlying structure. Notably, charges tend to diffuse or conduct more easily through a single grain but encounter a higher energy barrier when attempting to migrate across different grains. By overlaying the topographical grain information with the CPD maps (black dashed lines over CPD maps), we found that for lower potentials ($|V_{DC}|<10$ V), the charged region was confined to the central grain (#1) with no noticeable impact on the surrounding grains. However, at high potentials ($|V_{DC}|\approx 10$ V), multiple grains (#1 and #2) can be injected with charge. Therefore, the CPD changes more for the +8 V case than the +10 V case, since the injected charge remains trapped within the central grain (#1), while it distributes to the neighboring grain (#2) at 10 V. In Supplementary information Figure 11 the gradient of the topography and CPD images are shown, where grain boundaries and charge injected areas of influence are highlighted with more detail.

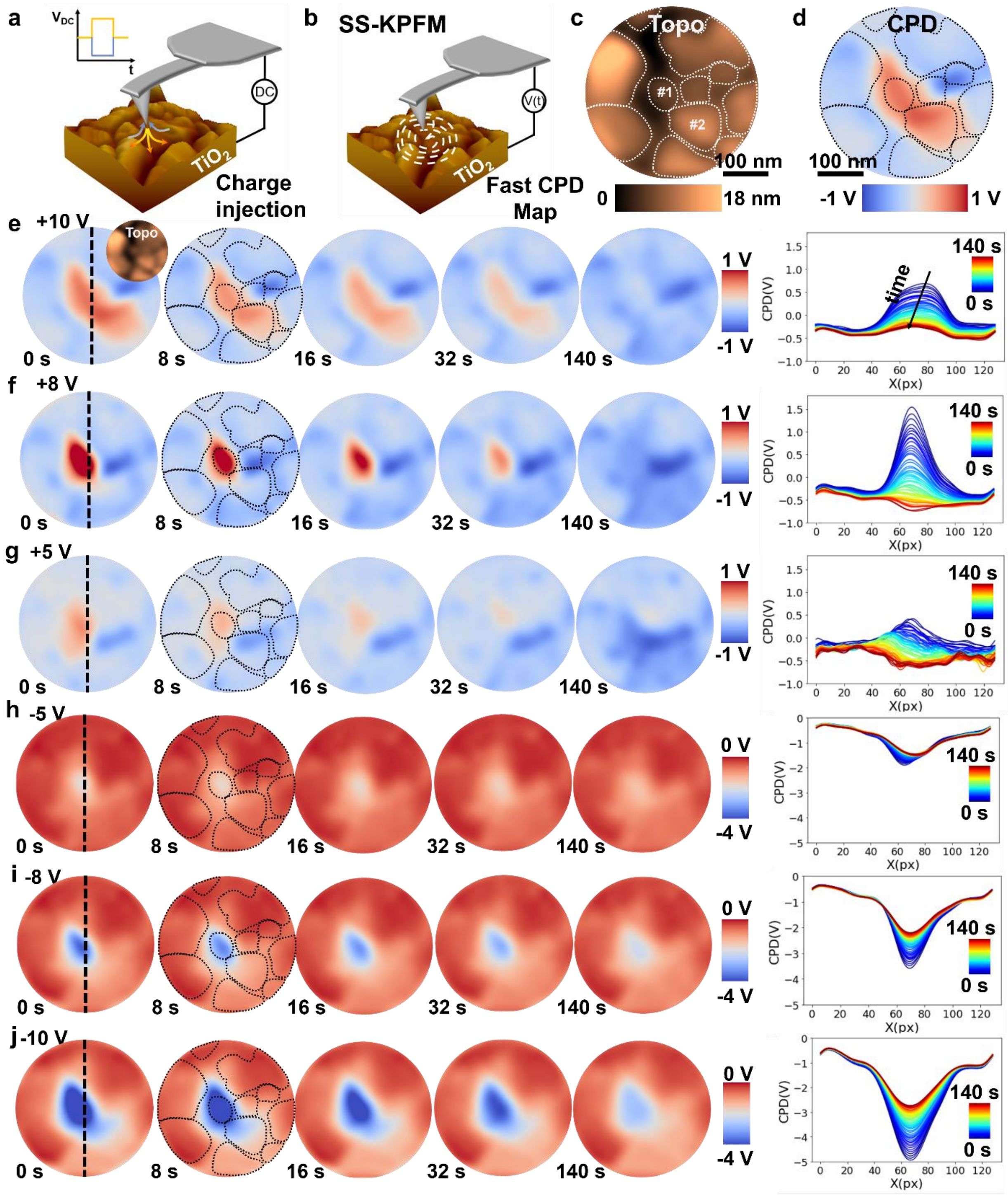

Supplementary Figure 10: Mapping charge injection and diffusion in polycrystalline TiO_2. a) A voltage pulse of 25 s duration and different amplitudes ranging from +10 V to -10 V is applied to the tip while in contact. b) Right after the pulse is complete, SS-KPFM is triggered, and surface charge is mapped at 2 seconds /frame. c) Topography map at *t*=0 s for the +10 V DC bias. d) CPD map at *t*=0 s for the +10 V DC bias. e-j) CPD maps and corresponding profiles along the dashed

black line for the cases of DC bias e) +10 V, f) +8 V, g) +5 V, h)-5 V, i) -8 V, j) -10 V. Inset shows the topography of the same region at t=0 s. All images have the same size.

Supplementary note 11. Gradient images for TiO_2 data set.

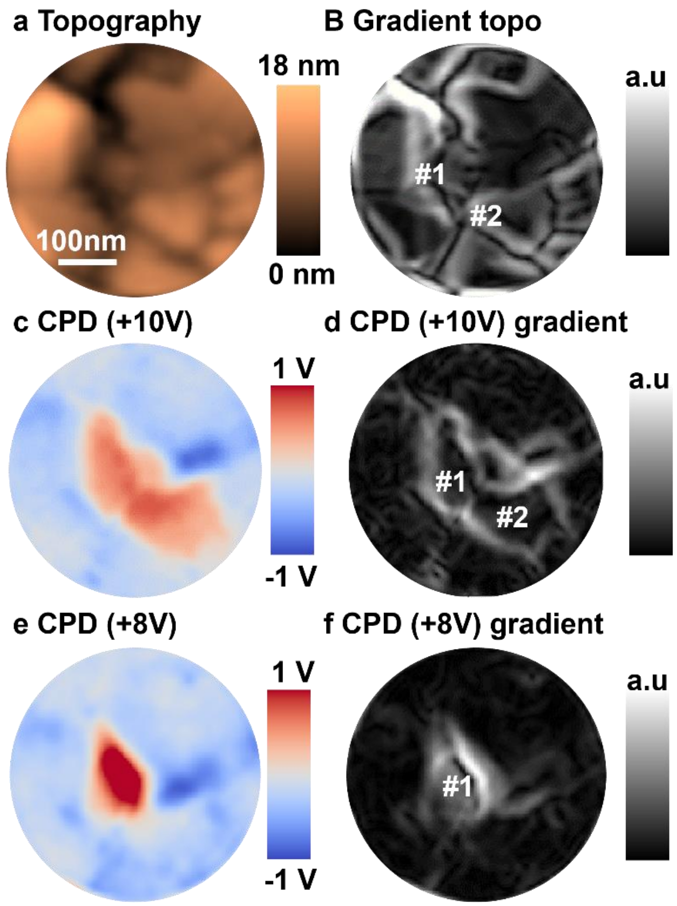

Supplementary Figure 11: a) Topography image. b) Gradient of the topography, which highlights the grain boundaries. c) and d) are CPD images after 10 V and 8 V bias pulses, respectively. e) and f) are the corresponding CPD gradient images, where the borders of the charge injected regions are highlighted. Gradient images are calculated as the square root of the sum of the squares of the gradient along *X* and *Y* directions. All images have the same size.

Supplementary note 12. Charge injection in one grain vs 2 grains simultaneously

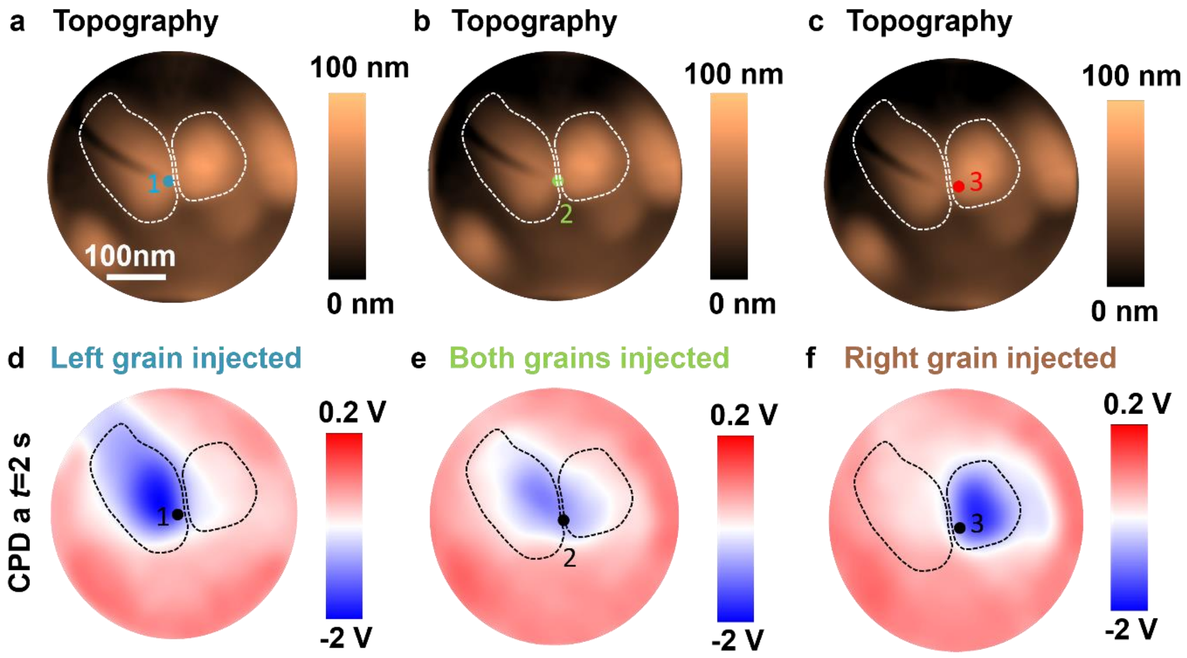

Supplementary Figure 12: Topography images corresponding to the charge injection at the left grain (a), at the grain boundary, touching both grains (b) and at the right grain (c). Corresponding CPD maps with charge injection at the left grain (d), at the grain boundary, touching both grains (e) and at the right grain (f). All images have the same size.

Supplementary note 13. TiO_2 structural characterization:

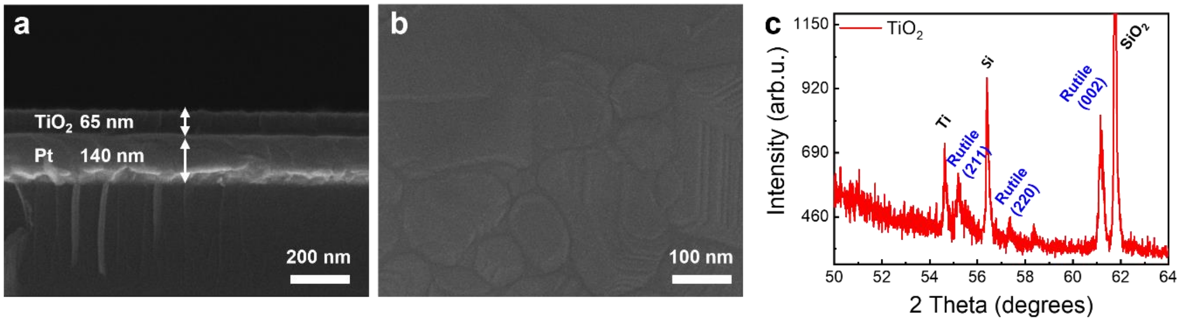

Supplementary Figure 13: (a, b) SEM images of the polycrystalline TiO_2 on the Pt/Ti/SiO_2/Si substrate: (a) cross-section and (b) surface. (c) XRD patterns of the polycrystalline TiO_2. The XRD pattern of the polycrystalline TiO_2 in Figure xc clearly shows (211), (220), and (002) peaks of rutile.

Supplementary references:

1 Sugawara, Y. *et al.* High potential sensitivity in heterodyne amplitude-modulation Kelvin probe force microscopy. *Applied Physics Letters* **100**, 223104 (2012).

2 Axt, A., Hermes, I. M., Bergmann, V. W., Tausendpfund, N. & Weber, S. A. Know your full potential: Quantitative Kelvin probe force microscopy on nanoscale electrical devices. *Beilstein journal of nanotechnology* **9**, 1809-1819 (2018).

3 Garrett, J. L. & Munday, J. N. Fast, high-resolution surface potential measurements in air with heterodyne Kelvin probe force microscopy. *Nanotechnology* **27**, 245705 (2016).

4 Garrett, J. L. *et al.* Real-time nanoscale open-circuit voltage dynamics of perovskite solar cells. *Nano letters* **17**, 2554-2560 (2017).

5 Du, Y. *et al.* The resistive switching in TiO2 films studied by conductive atomic force microscopy and Kelvin probe force microscopy. *AIP Advances* **3**, 082107 (2013).
