# Supplementary material for: High-speed mapping of surface charge dynamics using sparse scanning Kelvin probe force microscopy
Source: Nat Commun. 2023 Nov 8;14:7196. doi: 10.1038/s41467-023-42583-x (PMC10632481; doi:10.1038/s41467-023-42583-x)
Supplement: Supplementary file 3 — Description of Additional Supplementary Files [file 41467_2023_42583_MOESM3_ESM.pdf]

Description of Additional Supplementary Files:

File Name: Supplementary Movie 1 (SS-KPFM on LAOSTO for negative bias)
Description: Animation of the SS-KPFM dataset taken on LAO/STO for negative bias applied. from left to right: CPD Raw Data, GP reconstructed frame and corresponding profile over time.

File Name: Supplementary Movie 2 (SS-KPFM on LAOSTO for positive bias)
Description: Animation of the SS-KPFM dataset taken on LAO/STO for positive bias applied. from left to right: CPD Raw Data, GP reconstructed frame and corresponding profile over time.

File Name: Supplementary Movie 3 (SS-KPFM on TiO2 after +10V)
Description: Animation of the SS-KPFM dataset taken on TiO_2 after +10V bias applied. From left to right: CPD Raw Data, GP reconstructed frame and corresponding profile over time.

File Name: Supplementary Movie 4 (SS-KPFM on TiO2 after +8V)
Description: Animation of the SS-KPFM dataset taken on TiO_2 after +8V bias applied. From left to right: CPD Raw Data, GP reconstructed frame and corresponding profile over time.

File Name: Supplementary Movie 5 (SS-KPFM on TiO2 after +5V)
Description: Animation of the SS-KPFM dataset taken on TiO_2 after +5V bias applied. From left to right: CPD Raw Data, GP reconstructed frame and corresponding profile over time.

File Name: Supplementary Movie 6 (SS-KPFM on TiO2 after -5V)
Description: Animation of the SS-KPFM dataset taken on TiO_2 after -5V bias applied. From left to right: CPD Raw Data, GP reconstructed frame and corresponding profile over time.

File Name: Supplementary Movie 7 (SS-KPFM on TiO2 after -8V)
Description: Animation of the SS-KPFM dataset taken on TiO_2 after -8V bias applied. From left to right: CPD Raw Data, GP reconstructed frame and corresponding profile over time.

File Name Supplementary Movie 8 (SS-KPFM on TiO2 after -10V)
Description: Animation of the SS-KPFM dataset taken on TiO_2 after -10V bias applied. From left to right: CPD Raw Data, GP reconstructed frame and corresponding profile over time.
