# Supplementary figures and images for: High-speed mapping of surface charge dynamics using sparse scanning Kelvin probe force microscopy
Source: Nat Commun. 2023 Nov 8;14:7196. doi: 10.1038/s41467-023-42583-x (PMC10632481; doi:10.1038/s41467-023-42583-x)

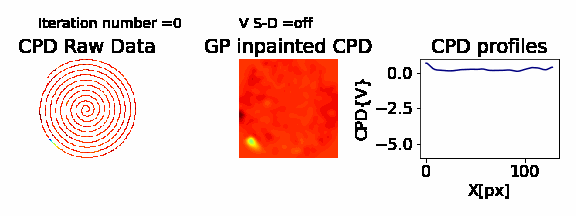

Supplement: Supplementary file 4 — Supplementay Movie 1 [file 41467_2023_42583_MOESM4_ESM.gif]

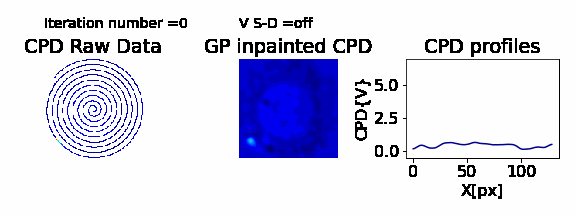

Supplement: Supplementary file 5 — Supplementay Movie 2 [file 41467_2023_42583_MOESM5_ESM.gif]

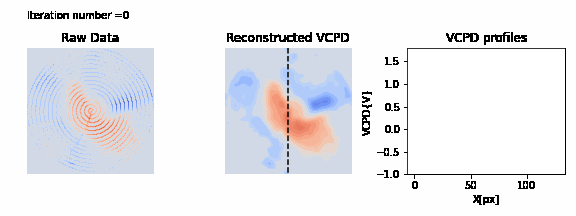

Supplement: Supplementary file 6 — Supplementay Movie 3 [file 41467_2023_42583_MOESM6_ESM.gif]

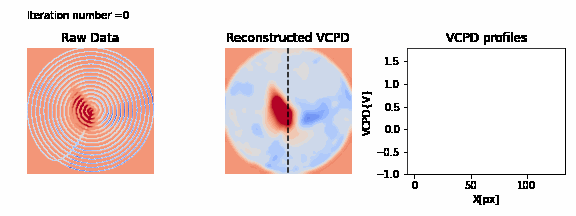

Supplement: Supplementary file 7 — Supplementay Movie 4 [file 41467_2023_42583_MOESM7_ESM.gif]

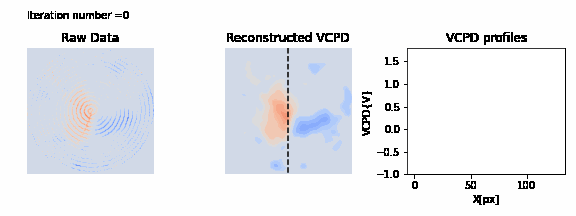

Supplement: Supplementary file 8 — Supplementay Movie 5 [file 41467_2023_42583_MOESM8_ESM.gif]

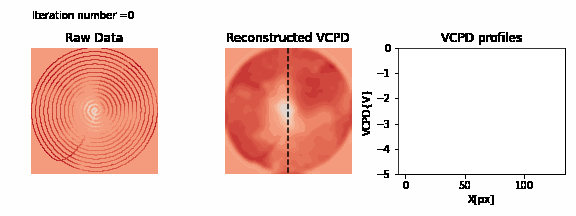

Supplement: Supplementary file 9 — Supplementay Movie 6 [file 41467_2023_42583_MOESM9_ESM.gif]

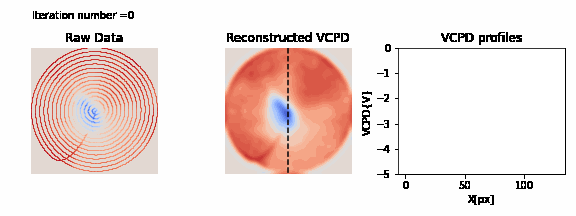

Supplement: Supplementary file 10 — Supplementay Movie 7 [file 41467_2023_42583_MOESM10_ESM.gif]

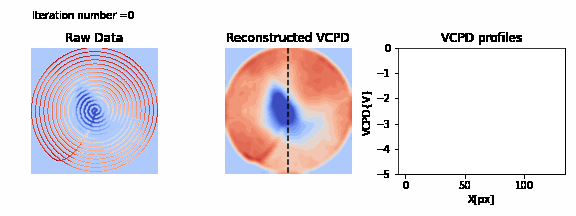

Supplement: Supplementary file 11 — Supplementay Movie 8 [file 41467_2023_42583_MOESM11_ESM.gif]
